# *Epi*-reevesioside F inhibits Na^+^/K^+^-ATPase, causing cytosolic acidification, Bak activation and apoptosis in glioblastoma

**DOI:** 10.18632/oncotarget.4429

**Published:** 2015-06-10

**Authors:** Jui-Ling Hsu, Fan-Lun Liu, Lih-Ching Hsu, Hsun-Shuo Chang, Wohn-Jenn Leu, Chia-Chun Yu, Wei-Ling Chang, Ih-Sheng Chen, Fan-Lu Kung, Jih-Hwa Guh

**Affiliations:** ^1^ School of Pharmacy, National Taiwan University, Taipei, Taiwan; ^2^ School of Pharmacy, College of Pharmacy, Kaohsiung Medical University, Kaohsiung, Taiwan; ^3^ Graduate Institute of Natural Products, College of Pharmacy, Kaohsiung Medical University, Kaohsiung, Taiwan; ^4^ The Division of Medicinal Chemistry, College of Pharmacy, The Ohio State University, Columbus, OH, USA

**Keywords:** Epi-reevesioside F, intracellular Na^+^ concentration, cytosolic acidification, mitochondrial dysfunction, bak activation

## Abstract

*Epi*-reevesioside F, a new cardiac glycoside isolated from the root of *Reevesia formosana*, displayed potent activity against glioblastoma cells. *Epi*-reevesioside F was more potent than ouabain with IC_50_ values of 27.3±1.7 *vs.* 48.7±1.8 nM (*P* < 0.001) and 45.0±3.4 *vs.* 81.3±4.3 nM (*P* < 0.001) in glioblastoma T98 and U87 cells, respectively. However, both *Epi*-reevesioside F and ouabain were ineffective in A172 cells, a glioblastoma cell line with low Na^+^/K^+^-ATPase α3 subunit expression. *Epi*-reevesioside F induced cell cycle arrest at S and G2 phases and apoptosis. It also induced an increase of intracellular concentration of Na^+^ but not Ca^2+^, cleavage and exposure of N-terminus of Bak, loss of mitochondrial membrane potential, inhibition of Akt activity and induction of caspase cascades. Potassium supplements significantly inhibited *Epi*-reevesioside F-induced effects. Notably, *Epi*-reevesioside F caused cytosolic acidification that was highly correlated with the anti-proliferative activity. In summary, the data suggest that *Epi*-reevesioside F inhibits Na^+^/K^+^-ATPase, leading to overload of intracellular Na^+^ and cytosolic acidification, Bak activation and loss of mitochondrial membrane potential. The PI3-kinase/Akt pathway is inhibited and caspase-dependent apoptosis is ultimately triggered in *Epi*-reevesioside F-treated glioblastoma cells.

## INTRODUCTION

Cardiac glycosides are a class of steroid-like compounds and have a long history in the treatment of cardiac diseases, such as congestive heart failure and arrhythmia. Inhibition of Na^+^/K^+^-ATPase by cardiac glycosides leads to intracellular Na^+^ overload which *in turn* drives Ca^2+^ influx into cells [1]. Appropriate increase of intracellular Ca^2+^ levels in cardiac cells results in an increase of contractility. However, intracellular Ca^2+^ overload may cause cell death since Ca^2+^ participates in signaling pathways of apoptosis and necrosis [2]. Because of the pivotal role in deciding cell fate, disturbance of Ca^2+^ homeostasis can be an anticancer strategy. It has been documented that cardiac glycosides, including bufotalin, oleandrin, ouabain and digoxin and their derivatives, induce anti-proliferative and apoptotic activities in cancer cells through an increase of intracellular Ca^2+^ levels [3-5]. Ca^2+^-independent signaling pathways also have been reported, such as oxidative stress [6], inhibition of NF-κB and AP-1 [7], an increase of FasL expression [7, 8], inhibition of topoisomerases [3], and down-regulation of pro-survival Bcl-2 family members [9] and proto-oncogene *c-myc* expression [10, 11].

Ion transport and intracellular pH are important in numerous cell functions, including cell membrane potential, cell volume, mitochondrial function, enzyme activity, DNA synthesis, cell proliferation, differentiation, oncogenesis, malignant transformation and metastasis [12, 13]. Aberrant regulation of H^+^ dynamics occurs in almost all tumors. Tumors, by using multiple ways for ion transport, create extracellular acid microenvironment while maintain alkaline intracellular pH, promoting survival, progression and metastasis [12-14]. Alkaline intracellular pH also contributes to chemotherapy resistance [15, 16]. Establishment of this self-defensive/anti-apoptotic mechanism is predominantly through anti-acidifying effect caused by hyperactivity of membrane-bound H^+^ extrusion transporters [17]. Many studies have suggested that intracellular acidification resulted from chemotherapeutic drugs is responsible for an early onset of malignant cell apoptosis [17-19]. Accordingly, H^+^ extrusion transporters are potential targets for anticancer strategy [17].

Recently, bioassay-guided fractionation of the root of *Reevesia formosana* resulted in the isolation of new cardiac glycosides with anti-proliferative and apoptotic activities in our work [11, 20, 21]. In this study, *Epi*-reevesioside F has been examined to display potent anti-proliferative activity in glioblastoma cells, leading to Bak activation, mitochondrial dysfunction and cell death. The signaling pathways related to ion transport and cytosolic acidification have been identified to demonstrate the anticancer potential of *Epi*-reevesioside F.

## RESULTS

### *Epi*-reevesioside F displays anti-proliferative activity against both T98 and U87 cells

**Figure 1:**
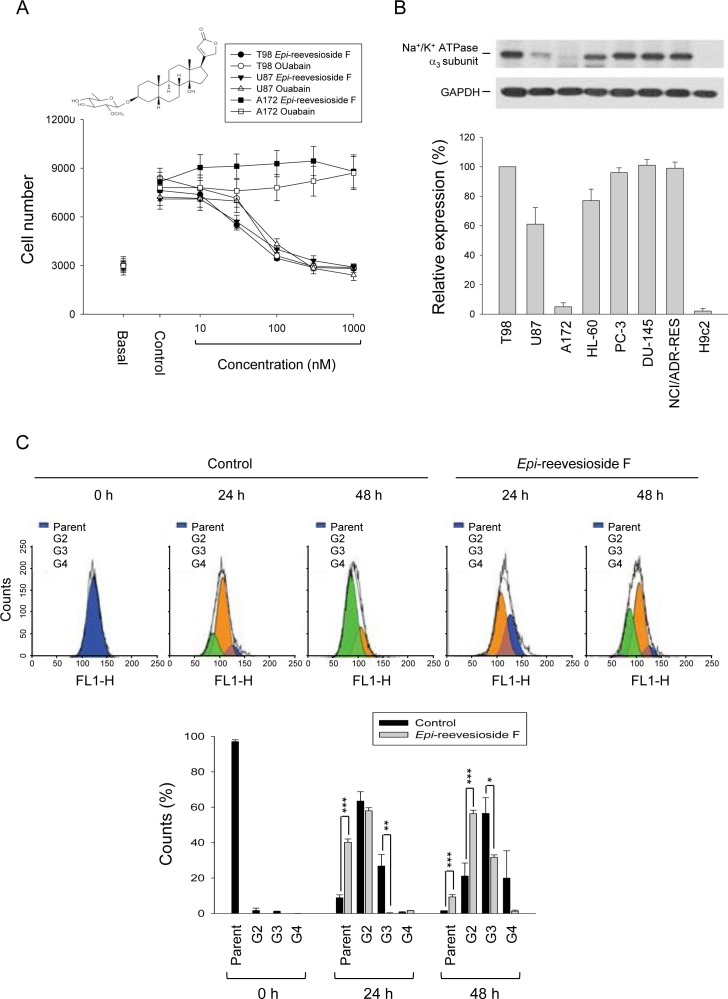
Effect of *Epi*-reevesioside F on cell proliferation and protein expression of Na^+^/K^+^-ATPase α3 subunit **(A)** The graded concentrations of *Epi*-reevesioside F and ouabain were added to the cells for 48 hours (T98 and A172 cells) or 72 hours (U87 cells). After the treatment, cell proliferation was determined using sulforhodamine B assay. **(B)** The cells were lysed for the detection of protein expression by Western blot analysis. The expression was quantified using computerized image analysis system ImageQuant (Amersham Biosciences). **(C)** T98 cells were incubated in the absence of presence of *Epi*-reevesioside F (100 nM) for the indicated time. After treatment, the cells were fixed and labeled with CFSE for flow cytometric analysis. Data are expressed as mean±SEM of three to five independent determinations. *P < 0.05, **P < 0.01 and ***P < 0.001 compared with the parent cells.

Both *Epi*-reevesioside F and ouabain induced concentration-dependent inhibition of cell proliferation in T98 and U87 cells using sulforhodamine B assay. *Epi*-reevesioside F was more potent than ouabain with IC_50_ values of 27.3±1.7 *vs.* 48.7±1.8 nM (*P* < 0.001) and 45.0±3.4 *vs.* 81.3±4.3 nM (*P* < 0.001) in T98 and U87 cells, respectively (Figure 1A). Both compounds were ineffective in A172 cells. Cardiac glycosides have been suggested to show distinct affinities toward Na^+^/K^+^-ATPase α-isoforms [22]. The detection of α-isoforms protein levels demonstrated that the expression of α3-subunit was much lower in A172 than that in T98 and U87 cells (Figure 1B). Several cell lines were also examined with varied expression levels of Na^+^/K^+^-ATPase α3-subunit (Figure 1B). *Epi*-reevesioside F-induced anti-proliferative effects were identified to have a high correlation (r^2^ = 0.96) with α3-subunit expressions. The inhibition of cell proliferation was further confirmed by CFSE assay. The data in Figure 1C demonstrated that the major population was the second and the third generation after seeding cells for 24 and 48 h, respectively. However, the population distribution significantly shifted to the parent and the second generation after the exposure to *Epi*-reevesioside F, respectively, confirming its anti-proliferative activity (Figure 1C).

### *Epi*-reevesioside F induces cell cycle arrest and differential apoptotic regulation between T98 and U87 cells

**Figure 2:**
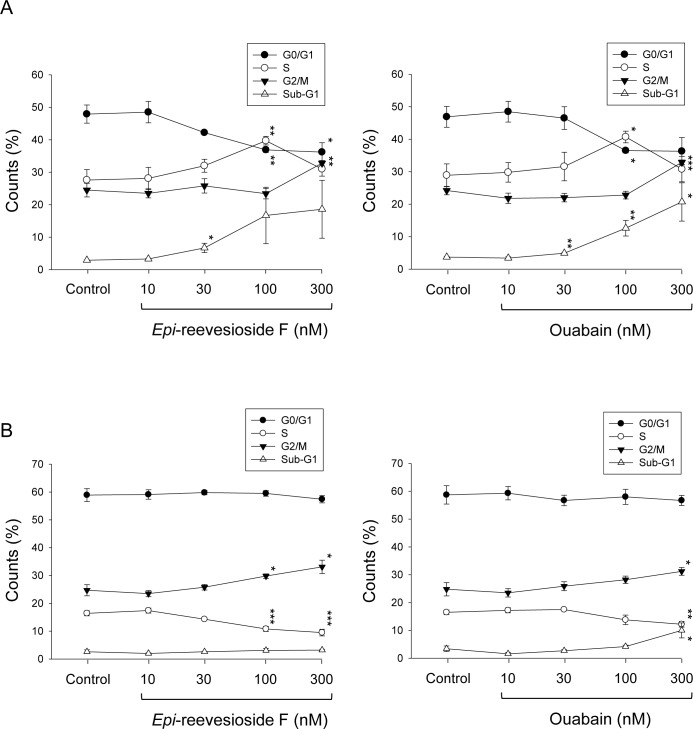
Effect of *Epi*-reevesioside F and ouabain on cell-cycle progression T98 **(A)** or U87 **(B)** cells were incubated in the absence or presence of *Epi*-reevesioside F or ouabain for 48 hours. After the treatment, the cells were harvested for the determination of cell cycle population using the detection of DNA content analyzed with FACScan and CellQuest software. Data are expressed as mean±SEM of three independent determinations. *P < 0.05, **P < 0.01 and ***P < 0.001 compared with the respective control.

Flow cytometric analysis of propidium iodide staining revealed that both *Epi*-reevesioside F and ouabain induced concentration-dependent arrest of cell cycle at S and G2/M phases associated with apoptosis in T98 cells (Figure 2A). In contrast, only a moderate increase of G2/M phase arrest was triggered in U87 cells (Figure 2B). DNA damage or DNA replication stress can activate several key kinases that induce cell cycle arrest predominantly at S and G2 phases to facilitate DNA repair [23]. Several downstream targets induced by DNA damage were examined, including Chk kinase, γ-H2A.X and replication protein A (RPA, protein complex associating with single-stranded DNA). The data showed that *Epi*-reevesioside F did not stimulate these cellular targets (Supplementary Figure 1) indicating that DNA was not a primary target for *Epi*-reevesioside F.

### *Epi*-reevesioside F increases intracellular Na^+^ concentrations

**Figure 3:**
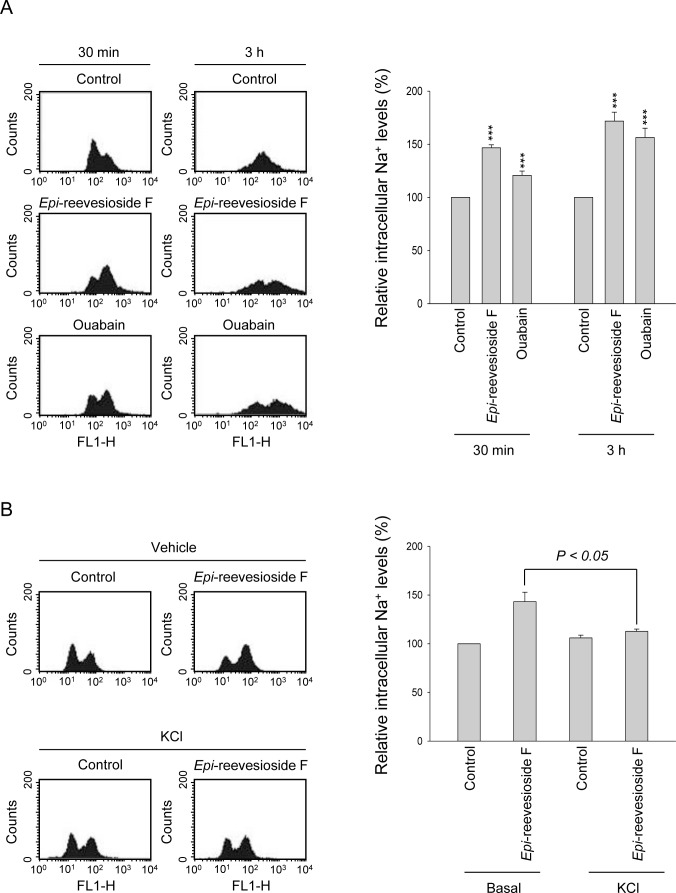
Effect of *Epi*-reevesioside F and ouabain on intracellular Na^+^ levels **(A)** T98 cells were incubated in the absence or presence of *Epi*-reevesioside F (100 nM) or ouabain (100 nM) for the indicated time, or **(B)** T98 cells were incubated in the absence or presence of *Epi*-reevesioside F (100 nM) with or without extracellular potassium supplements (10.7 mM) for 3 hours. After the treatment, the intracellular Na^+^ levels were examined using flow cytometric analysis. Data are expressed as mean±SEM of three to five independent determinations. ***P < 0.001 compared with the respective control.

**Figure 4:**
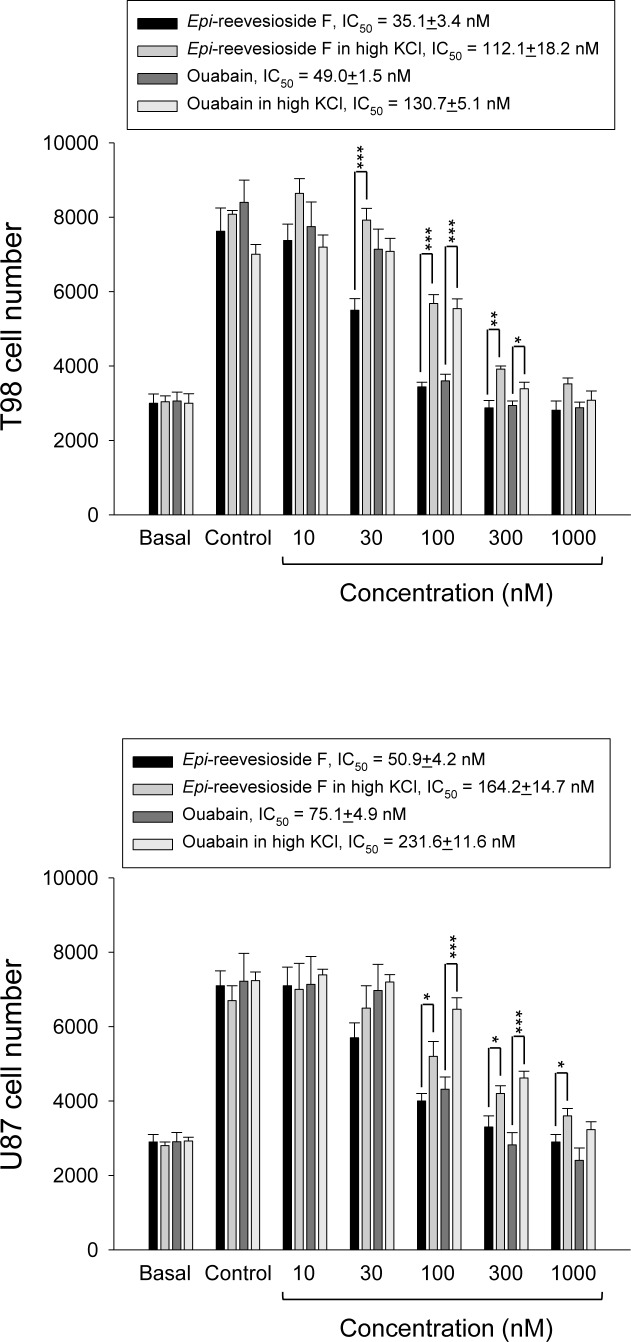
Effect of potassium supplements on cardiac glycoside-induced anti-proliferation T98 or U87 cells were incubated in the absence or presence of *Epi*-reevesioside F or ouabain for 48 hours (T98) or 72 hours (U87) with or without extracellular potassium supplements (10.7 mM). After the treatment, cell proliferation was determined using sulforhodamine B assay. Data are expressed as mean±SEM of four to five independent determinations. *P < 0.05, **P < 0.01 and ***P < 0.001 compared with cardiac glycoside alone.

Several lines of evidence have suggested that cells may accumulate in G1/S and G2 phases under hyperosmotic stress [24, 25]. Because *Epi*-reevesioside F induced cell cycle arrest at both S and G2/M phases and inhibited cell proliferation, the intracellular Na^+^ concentrations were examined. The data demonstrated that both *Epi*-reevesioside F and ouabain caused significant increases of intracellular Na^+^ concentrations (Figure 3A), which were almost completely abolished by extracellular potassium supplements (Figure 3B). Furthermore, both *Epi*-reevesioside F- and ouabain-induced inhibition of cell proliferation in T98 and U87 cells was significantly rescued by extracellular potassium supplements (Figure 4).

The rise of intracellular Na^+^ concentrations induced by inhibition of Na^+^/K^+^-ATPase may drive the influx of Ca^2+^ through Na^+^/Ca^2+^ exchange system [1]. Intracellular Ca^2+^ overload can lead to cell death because Ca^2+^ participates in a variety of cell death programs [2]. Flow cytometric analysis of fluo-3 staining showed that both *Epi*-reevesioside F and ouabain did not modify intracellular Ca^2+^ concentrations (Supplementary Figure 2).

### *Epi*-reevesioside F decreases intracellular pH and inhibits cell proliferation

**Figure 5:**
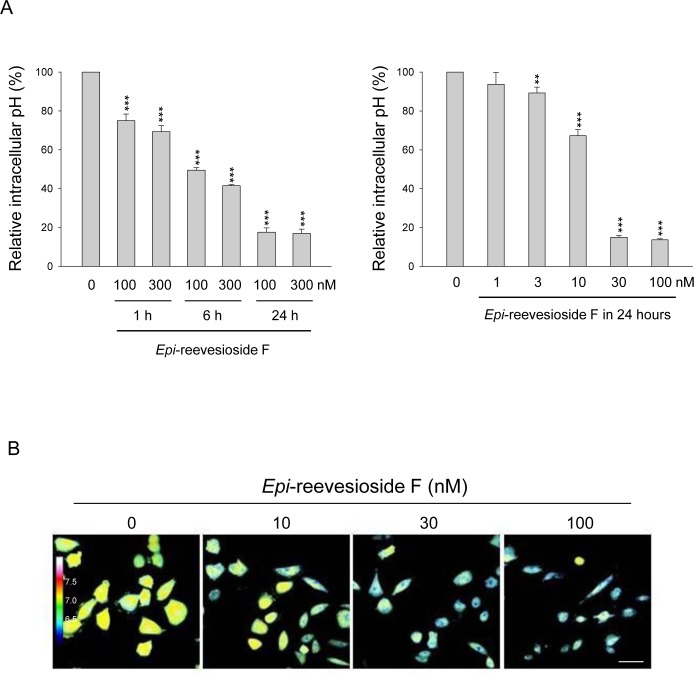
Effect of *Epi*-reevesioside F on intracellular pH T98 cells were incubated in the absence or presence of *Epi*-reevesioside F for the indicated time **(A)** or for 24 hours **(B).** After the treatment, the intracellular pH was determined using flow cytometric analysis **(A)**, or confocal immunofluorescence microscopic examination **(B).** Data are expressed as mean±SEM of three independent determinations. **P < 0.01 and ***P < 0.001 compared with the respective control. *Bar*, 50 μm.

The relationship between changes in intracellular pH and cell proliferation has attracted attention in anticancer researches. It has been suggested that cytoplasmic acidification may blunt proliferation and promote apoptosis in various cancer cell lines through a decrease of protein synthesis and influence of signaling pathways which govern cell survival [26, 27]. Flow cytometric analysis of fluorescent dye seminaphtharhodafluor-1 staining showed that *Epi*-reevesioside F induced time- and concentration-dependent intracellular acidification in T98 cells (Figure 5A). Confocal microscopic examination also demonstrated similar intracellular pH-lowering effect (Figure 5B). The correlation between intracellular pH and cell proliferation was determined with a high correlation coefficient (r^2^ = 0.98, Supplementary Figure 3), revealing that intracellular acidity could be a key factor in *Epi*-reevesioside F-induced effect.

Sodium/hydrogen exchangers (NHEs) constitute a large family of integral membrane protein transporters responsible for counter transport of H^+^ and Na^+^. The expression of NHE isoform 1 (NHE1) is ubiquitous. NHE1 activation is sensitive to intracellular acidic pH and is rapidly induced upon a drop of cytosolic pH [28]. The effect of *Epi*-reevesioside F on NHE1 activity was studied using immunoprecipitation analysis because the binding of 14-3-3 to NHE1 has been identified to be associated with the activation of Na^+^/H^+^ exchange [29]. As a result, *Epi*-reevesioside F significantly increased the association between NHE1 and 14-3-3, indicating an increase of NHE1 activity (Supplementary Figure 4). The data support the intracellular acidification to *Epi*-reevesioside F action.

### *Epi*-reevesioside F induces mitochondrial damage and caspase-dependent apoptosis

**Figure 6:**
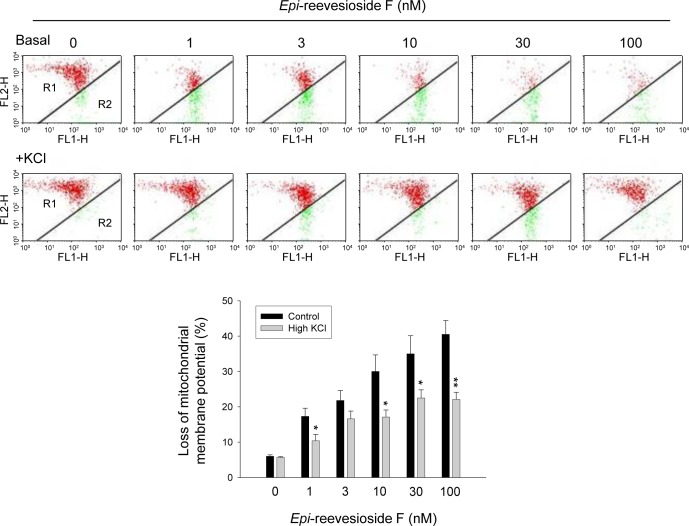
Effect of *Epi*-reevesioside F on mitochondrial membrane potential (ΔΨ_m_) T98 cells were incubated in the absence or presence of *Epi*-reevesioside F for 24 hours with or without extracellular potassium supplements (10.7 mM). After the treatment, the cells were incubated with JC-1 for the detection of ΔΨ_m_ using flow cytometric analysis. Data are expressed as mean±SEM of four independent determinations. *P < 0.05 and **P < 0.01 compared with *Epi*-reevesioside F alone.

Intracellular acidification has been suggested to be responsible for mitochondrial dysfunction and both caspase-dependent and -independent apoptosis under several apoptotic stimuli [30]. Furthermore, a decrease of intracellular pH may stimulate several acid DNases to promote cell death in many apoptotic conditions [31]. Mitochondrial membrane potential (ΔΨ_m_) was determined using JC-1 staining. The data demonstrated that *Epi*-reevesioside F induced a concentration-dependent increase of JC-1 monomers (green fluorescence) indicating a loss of ΔΨ_m_ and mitochondrial damage (Figure 6). The effect was significantly inhibited by extracellular potassium supplements (Figure 6).

**Figure 7:**
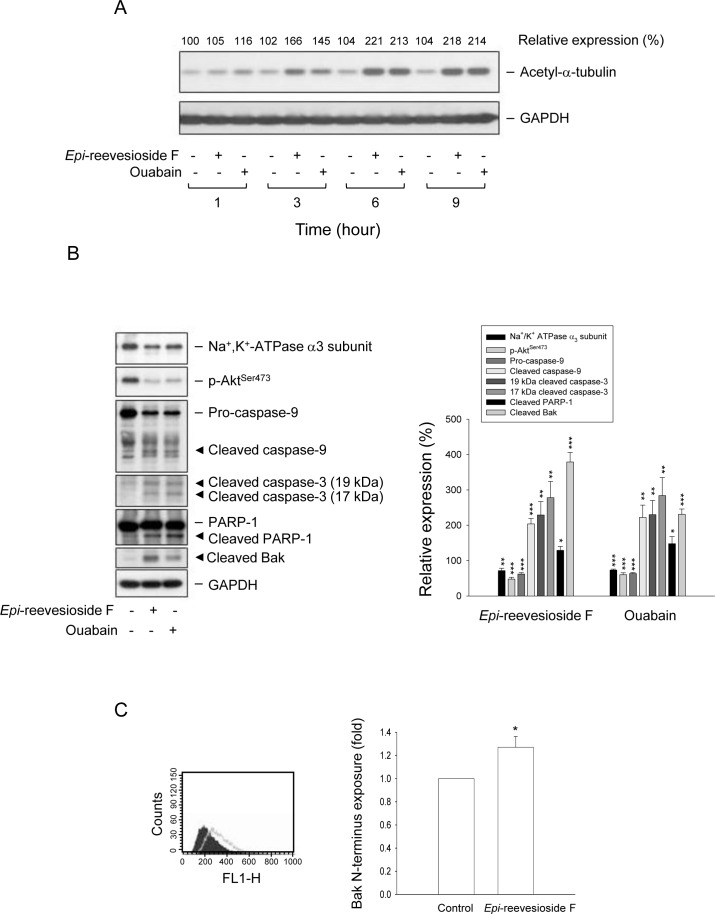
Effect of *Epi*-reevesioside F and ouabain on several protein expressions T98 cells were incubated in the absence or presence of *Epi*-reevesioside F (100 nM) or ouabain (100 nM) for the indicated time **(A)**, or for 48 hours **(B** and **C)**. After the treatment, the cells were harvested for the detection of protein expression using Western blot analysis (**A** and **B**) or flow cytometric analysis **(C).** Data are expressed as mean±SEM of three independent determinations. *P < 0.05, **P < 0.01 and ***P < 0.001 compared with the respective control.

In cells of neural and non-neural origins, tubulin may form a complex with Na^+^/K^+^-ATPase to regulate enzyme activity. Acetylated tubulin has been identified to be associated with Na^+^/K^+^-ATPase and to block the catalytic activity of the pump [32]. Both *Epi*-reevesioside F and ouabain induced a rapid and time-dependent increase of α-tubulin acetylation (Figure 7A). Further identification showed that both cardiac glycosides caused a profound decrease of protein expression of Na^+^/K^+^-ATPase α3 subunit and phosphorylated Akt (Ser473), and induced activation of caspase-9 and -3 and cleavage of PARP-1, a caspase-3 substrate (Figure 7B). Bak, a pro-apoptotic Bcl-2 family member, localizes to mitochondria and serves as an apoptotic inducer. After proteolysis, the cleaved Bak reveals a zinc binding site which regulates Bak activity through dimerization [33]. As a result, both *Epi*-reevesioside F and ouabain induced a dramatic increase of cleaved Bak formation (Figure 7B). Moreover, flow cytometric analysis showed that *Epi*-reevesioside F significantly enhanced the exposure of N terminus of Bak, suggesting an increase of Bak activity (Figure 7C).

## DISCUSSION

Several components have been isolated from the root of *Reevesia formosana*, including *Epi*-reevesioside F, reevesioside A, reevesioside D and reevesioside F. The anti-proliferative activities of these components against glioblastoma cell lines, A172, T98 and U87, were determined. The data demonstrated that all components showed little activities in A172 cells. In contrast, *Epi*-reevesioside F, reevesioside A and reevesioside F showed potent activities against T98 and U87 cells. Notably, *Epi*-reevesioside F exhibited higher activities (about two times) than ouabain, while reevesioside A and reevesioside F were equipotent to ouabain, in these two cell lines. Reevesioside D was ineffective in all cell lines. Therefore, *Epi*-reevesioside F has been selected for anti-glioblastoma study. Na^+^/K^+^-ATPase serves as a multifunctional signal transducer and integrator and is responsible for maintaining resting potential and in regulating cellular volume, contractility, adhesion, inflammation and apoptosis [11, 21, 34, 35]. The aberrant expression and activity of Na^+^/K^+^-ATPase have been implicated in the progression of several types of aggressive cancers. α1 and α3 subunits of Na^+^/K^+^-ATPase are frequently overexpressed in colorectal cancer, non-small cell lung cancer, melanomas and glioblastomas [36-38]. Targeting α1 and α3 subunits has been suggested to trigger both apoptosis- and non-apoptosis related death in cancer cells [11, 21, 36-38] and in cells with multidrug-resistant phonotype [39]. It suggests that Na^+^/K^+^-ATPase is a crucial target for development of anticancer agents. *Epi*-reevesioside F displayed potent anti-glioblastoma activity and Na^+^/K^+^-ATPase α3 subunit was important in determining this activity. Moreover, *Epi*-reevesioside F induced profound down-regulation of the α3 subunit. It has been suggested that both endogenous and exogenously applied cardiac glycosides can induce internalization and lysosomal degradation of the ligated Na^+^/K^+^-ATPase and eliminate the pump through a conformational change of the protein itself [40]. The data further supported that Na^+^/K^+^-ATPase α3 subunit served as a primary target of *Epi*-reevesioside F. In addition to α3 subunit, the protein levels of other subunits, including α1, α2 and β1, also have been examined. The data demonstrated varied levels of subunit expressions in different cell lines (supplementary figure 5). Notably, A172 cells expressed high levels of α1, α2 and β1 subunits. However, *Epi*-reevesioside F did not show activity on the inhibition of A172 proliferation. Similar low effect but high expression of α1 subunit expression was obtained in H9c2 cells to *Epi*-reevesioside F action. Overall, *Epi*-reevesioside F-induced anti-proliferative effects in these cell lines were highly correlated to the expression of α3 subunit but not α1 or α2 subunit.

Microtubules are highly dynamic polymers involved in a variety of cellular processes, such as cell division, differentiation and signal transduction. α-tubulin and β-tubulin are two major members of tubulin superfamily making up microtubule. Tubulin acetylation, a post-translational modification, may help recruit molecular chaperons and regulate client proteins involved in cell proliferation and apoptosis [41]. Tubulin can also form a complex with Na^+^/K^+^-ATPase to orchestrate the pump activity. However, acetylated tubulin can bind to Na^+^/K^+^-ATPase and blunt its catalytic activity [32]. Moreover, tubulin acetylation is correlated to cell apoptosis. A number of small molecule inhibitors in inhibiting histone deacetylase 6 have been reported to induce tubulin acetylation and to display anti-proliferative and apoptotic activities [42]. *Epi*-reevesioside F induced tubulin acetylation which correlated with the inhibition of Na^+^/K^+^-ATPase activity and could contribute to the anti-glioblastoma activity.

*Epi*-reevesioside F induced cell cycle arrest at S and G2/M phases in T98 cells and that at G2/M phase in U87 cells. It has been reported that proscillaridin A, a cardiac glycoside inhibitor of Na^+^/K^+^-ATPase, induced G2/M arrest in glioblastoma cell lines [43]. Bufotalin, a cardiac glycoside secreted by a number of toad species, also induced G2/M arrest through down-regulation of several cell cycle regulators including aurora A, Cdc25, cyclin-dependent kinase 1 and cyclin A, and up-regulation of p53 and p21 in hepatocellular carcinoma HepG2 cells [4]. After further examination, *Epi*-reevesioside F neither modified protein expressions of cell cycle regulators (data not shown) nor induced DNA damage during the initiation of cellular stress in glioblastoma cells. It is noteworthy that several types of cells exposed to hyperosmolality undergo growth arrest at S and G2/M phases of cell cycle [24, 25]. A number of proteins and kinases are involved in hyperosmotic cell cycle delay, including GADD45, GADD153, extracellular signal-regulated kinases 1 and 2, c-Jun N-terminal kinase and p38 mitogen-activated protein kinase [25, 44]. *Epi*-reevesioside F resulted in a significant increase of intracellular Na^+^ concentrations and a decrease of cell size indicating the induction of hyperosmotic stress. It could explain the cell cycle arrest and inhibition of cell proliferation in glioblastoma cells. Notably, phosphoinositide 3-kinase (PI3-kinase)/Akt pathway is crucial in determining the cell fate under hyperosmotic stress. The activation of PI3-kinase/Akt has been suggested to prevent mild hyperosmolality-induced apoptotic stress in Madin-Darby canine kidney cells [45]. On the contrary, the hyperosmolar stress can down-regulate PI3-kinase activity and induce apoptosis in several types of cells [46]. The present work showed that *Epi*-reevesioside F caused the decrease of Akt phosphorylation on Ser-473, indicating an inhibition of Akt activity. The data reveal that inhibition of PI3-kinase/Akt pathway may confer *Epi*-reevesioside F-induced anti-glioblastoma activity in a hyperosmolar stress.

Several lines of evidence showing that hyperosmotic stress leads to cytosolic acidification followed by mitochondrial dysfunction and activation of apoptotic pathway. During the suicidal program, Ca^2+^ entry through Ca^2+^-permeable cation channels or exchangers may decrease mitochondrial integrity, activating several proteinases and inducing cell shrinkage [47]. The data demonstrated that *Epi*-reevesioside F induced a rapid and significant increase of intracellular Na^+^ concentrations and cytosolic acidification followed by the loss of mitochondrial membrane potential which was significantly rescued by extracellular potassium supplements. However, *Epi*-reevesioside F did not modify the intracellular Ca^2+^ concentrations. The data indicate that the ion transport and cytosolic acidification, but not Ca^2+^ mobilization, contribute to mitochondria-involved apoptotic cell death in glioblastoma cells. Several anticancer approaches have been proposed to induce intracellular acidification. The rationale is based on the consideration that under conditions of cytosolic acidification, the cells are susceptible to DNA degradation by acid-activated deoxyribonuclease (DNase) although Ca^2+^/Mg^2+^-dependent endonucleases dominate DNA digestion in several apoptotic conditions [48]. Furthermore, it has been suggested that DNA digestion in cell apoptosis frequently is not related to alterations in intracellular Ca^2+^ levels but correlates with intracellular acidification [49]. A variety of cancer chemotherapeutic drugs have been discovered to induce apoptosis on the basis of intracellular acidification and activation of acid-activated DNase II [49]. These studies reveal that targeting the mechanisms of intracellular pH regulation can be a potential anticancer strategy. To this end, *Epi*-reevesioside F which inhibits Na^+^/K^+^-ATPase, leading to changes in ion transport and intracellular acidification may be a potential candidate for further development.

Bcl-2 family proteins including pro-apoptotic (e.g., Bax, Bak, Bad and Bid) and anti-apoptotic members (e.g., Bcl-2, Bcl-xL and Mcl-1) govern mitochondrial outer membrane permeability. Many Bcl-2 family proteins bind themselves and each other to form homodimers or heterodimers. Increasing lines of evidence suggest that changes in cytosolic pH may regulate the functions of Bcl-2 family members [50]. Dimerization of Bcl-2 family proteins can be promoted under lower pH through a decreased rate of dimer dissociation [51]. Moreover, mitochondrial proton transporters and proton circuit can be affected by variations in cytosolic pH [52]. Of note, apoptosis induced by cytosolic acidification can be either caspase-dependent or caspase-independent [30, 50]. In this regard, *Epi*-reevesioside F induced a significant loss of mitochondrial membrane potential and a dramatic increase of cleaved Bak formation and its exposure of N terminus suggesting a profound increase of Bak activity [33]. In addition, caspase-dependent apoptosis was activated to *Epi*-reevesioside F action. Bak is constitutively expressed in mitochondrial outer membrane. Bak and Bax form mitochondrial apoptosis-induced channel and, furthermore, Bak interacts and enhances the opening of mitochondrial voltage-dependent anion channel, making mitochondrial outer membrane permeable to pro-apoptotic proteins, such as cytochrome *c* and Smac/DIABLO [53, 54]. It is essential in coordinating apoptosis induced by various cancer chemotherapeutic drugs in a variety of cancers, including temozolomide in the treatment of glioblastoma multiforme [55]. The data suggest that Bak activation may enhance *Epi*-reevesioside F-induced anti-glioblastoma activity through mitochondrial apoptosis pathway.

Taken together, the data suggest that *Epi*-reevesioside F can potently inhibit Na^+^/K^+^-ATPase, leading to overload of intracellular Na^+^, changes in ion transport and cytosolic acidification which *in turn* result in Bak activation and a loss of mitochondrial membrane potential. The PI3-kinase/Akt pathway is inhibited and caspase-dependent apoptosis is ultimately triggered in *Epi*-reevesioside F-treated glioblastoma cells. The data suggest that *Epi*-reevesioside F can be a potential candidate for anti-glioblastoma development.

## MATERIALS AND METHODS

### Materials

DMEM and fetal bovine serum (FBS) were obtained from GIBCO/BRL Life Technologies (Grand Island, NY). Antibodies to caspase-3, PARP-1, α-tubulin, and anti-mouse and anti-rabbit IgGs were obtained from Santa Cruz Biotechnology, Inc. (Santa Cruz, CA). Antibodies to acetyl-α-tubulin^Lys40^, p-Akt^Ser473^ and p-Akt^Thr308^ were from Cell Signaling Technologies (Boston, MA). Ouabain, propidium iodide (PI), sulforhodamine B, RNase, trichloroacetic acid (TCA), Triton X-100, phenylmethylsulfonyl fluoride, leupeptin, aprotinin, sodium fluoride, sodium orthovanadate and proteinase K were obtained from Sigma-Aldrich (St. Louis, MO). Fluo-3/AM, JC-1 and carboxyfluorescein succinimidyl ester (CFSE) were from Molecular Probes Inc. (Eugene, OR, USA). *Epi*-reevesioside F was isolated from the root of *Reevesia formosana*. Purification and identification of *Epi*-reevesioside F were published elsewhere [20].

### Cell lines and cell culture

A172, T98 and U87, three human cell lines derived from glioblastoma, were from American Type Culture Collection (Rockville, MD). Cells were cultured in DMEM supplemented with 10% heat-inactive FBS (*v/v*), penicillin (100 units/ml) and streptomycin (100 μg/ml). Cultures were maintained in a 37°C** incubator with 5% CO_2_.

### Sulforhodamine B assay

Cells were seeded in 96-well plates in medium with 10% FBS. After 24 hours, cells were fixed with 10 % TCA to represent cell population at the time of compound addition (T_0_). After additional incubation of 0.1% dimethylsulfoxide (DMSO) or the indicated compound for 48 hours in A172 and T98 cells (doubling time between 24 and 30 hours) or 72 hours in U87 cells (doubling time, 60 hours), cells were fixed with 10 % TCA and sulforhodamine B at 0.4 % (w/v) in 1 % acetic acid was added to stain cells. Unbound sulforhodamine B was washed out by 1 % acetic acid. Sulforhodamine B bound cells were solubilized with 10 mM Trizma base. The absorbance was read at a wavelength of 515 nm. Using the following absorbance measurements, such as time zero (T_0_), control growth (C), and cell growth in the presence of the indicated compound (Tx), the percentage growth was calculated at each of the compound concentrations levels. Percentage growth inhibition was calculated as: [1−(Tx-T_0_)/(C-T_0_)] × 100%. Growth inhibition of 50% (IC_50_) is determined at the compound concentration which results in 50% reduction of total protein increase in control cells during the compound incubation.

### Cell proliferation assay by CFSE labeling

CFSE was dissolved in DMSO to constitute a storage solution of 10 mM and kept at −80°C until use. The cells were adjusted to a density of 10^6^ cells/ml and were treated with CFSE at a final concentration of 10 μM. After incubation at 37°C for 10 minutes, DMEM with 10% FCS was added. Tubes were placed in ice for 5 minutes and then washed. After centrifugation, the cells were seeded in DMEM with 10% FCS for the indicated times at 37°C in 5% CO_2_/95% air. After the treatment, the fluorescence intensity was determined by flow cytometric analysis (Becton Dickinson, Mountain View, CA).

### Flow cytometric assay of PI staining

After the exposure to the indicated agent, cells were harvested by trypsinization, fixed with 70 % (*v/v*) alcohol at 4°C for 30 minutes and washed with PBS. The cells were centrifuged and resuspended with 0.5 ml PI solution containing Triton X-100 (0.1%, *v/v*), RNase (100 μg/ml) and PI (80 μg/ml). DNA content was analyzed with the FACScan and CellQuest software (Becton Dickinson, Mountain View, CA).

### Measurement of intracellular Ca^2+^ concentrations

Cells were pre-incubated with fluo-3/AM (2.5 μM) for 30 minutes. The cells were washed twice and incubated in fresh medium. Vehicle (0.1% DMSO), *Epi*-reevesioside F or ouabain was added to the cells for the indicated times. The intracellular Ca^2+^ concentrations were determined by flow cytometric analysis (Becton Dickinson, Mountain View, CA).

### Measurement of ΔΨ_m_

JC-1, a mitochondrial dye staining mitochondria in living cells in a membrane potential-dependent fashion, was used to determine ΔΨ_m_. Cells were treated with or without *Epi*-reevesioside F. Thirty minutes before the termination of incubation, the cells were incubated with JC-1 (final concentration of 2 μM) at 37°C for 30 minutes. The cells were finally harvested and the accumulation of JC-1 was determined using flow cytometric analysis (Becton Dickinson, Mountain View, CA).

### Measurement of intracellular Na^+^ concentrations

After the treatment, the cells were incubated with CoroNa Green (1 μM) for 45 minutes. The cells were washed twice and suspended in PBS. The intracellular Na^+^ concentrations were determined by flow cytometric analysis (Becton Dickinson, Mountain View, CA).

### Detection of intracellular acidification

After the treatment, the cells were incubated with seminaphtharhodafluor-1 (1 μM) for 45 minutes. The cells were washed twice and suspended in PBS. The fluorescence emission in channels FL2 and FL3 was analyzed with a flow cytometer. During the elaboration of the data, a ratio of the fluorescence emissions and the mean fluorescence ratio were calculated. Furthermore, isolated T98 cells were recorded in an open chamber slide and were analyzed by a confocal laser microscopic system (Leica TCS SP2).

### Western blotting

After the treatment, the cells were harvested by trypsinization, centrifuged and lysed in 0.1 ml of lysis buffer (10 mM Tris-HCl pH 7.4, 150 mM NaCl, 1mM EGTA, 1 % Triton X-100, 1 mM phenylmethylsulfonyl fluoride, 10 μg/ml leupeptin, 10 μg/ml aprotinin, 50 mM sodium fluoride and 100 μM sodium orthovanadate). For Western blot analysis, 40 μg proteins were separated by electrophoresis in a 12% polyacrylamide gel and transferred to a PVDF membrane. After one hour incubation at room temperature in PBS/5% non-fat milk, the membrane was washed with PBS/0.1% Tween 20 for another 1 hour and overnight incubated with the indicated antibody at 4°C. After three washings with PBS/0.1% Tween 20, the anti-mouse or anti-rabbit IgG (dilute 1:4000) was applied to the membranes for 1 hour at room temperature. The membranes were washed with PBS/0.1% Tween 20 for 1 hour and the detection of signal was performed with an enhanced chemiluminescence detection kit (Amersham, Buckinghamshire, UK).

### Flow cytometric assay of exposed N terminus of Bak

After the treatment, the cells were harvested by trypsinization, fixed with 4% (v/v) paraformaldehyde (pH=7.4) at 4°C for 5 minutes and washed with PBS. After centrifugation, cells were permeabilized with 0.01% saponin/PBS and incubated with anti-Bak antibody (N terminus) or IgG for 1 hour at 4°C. The cells were then washed and incubated with FITC-labeled anti-mouse secondary antibody for another 1 hour at 4°C. Cells were washed and re-suspended in PBS for the flow cytometric analysis (Becton Dickinson, Mountain View, CA).

### Data analysis

Data are presented as the mean±SEM for the indicated number of separate experiments. Statistical analysis of data for multiple groups is performed with one-way analysis of variance (ANOVA). Student's *t*-test is applied for comparison of two groups. *P*-values less than 0.05 are statistically considered significant.

## SUPPLEMENTARY MATERIAL FIGURES


