# Supplementary material for: Epi-reevesioside F inhibits Na+/K+-ATPase, causing cytosolic acidification, Bak activation and apoptosis in glioblastoma
Source: Oncotarget. 2015 Jun 10;6(27):24032–46. doi: 10.18632/oncotarget.4429 (PMC4695168; doi:10.18632/oncotarget.4429)
Supplement: Supplementary file 1 [file oncotarget-06-24032-s001.pdf]

Epi-reevesioside F inhibits Na^+/K^+-ATPase, causing cytosolic acidification, Bak activation and apoptosis in glioblastoma

Supplementary Material

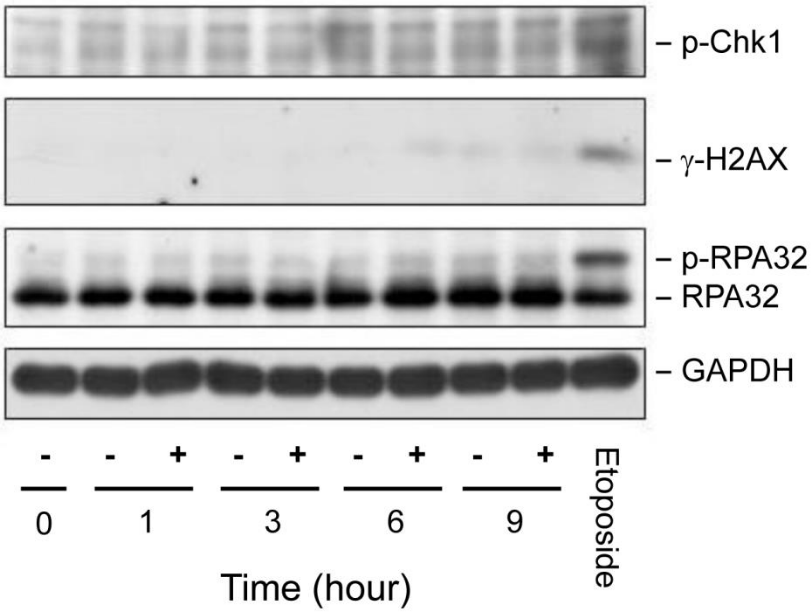

Supplementary figure 1: Effect of *Epi*-reevesioside F on DNA damage-related protein expressions. T98 cells were incubated in the absence or presence of *Epi*-reevesioside F (100 nM) for the indicated time. After the treatment, the cells were harvested for the detection of protein expression using Western blot analysis. Data are the representative of two independent tests. Etoposide (30 mM), positive control.

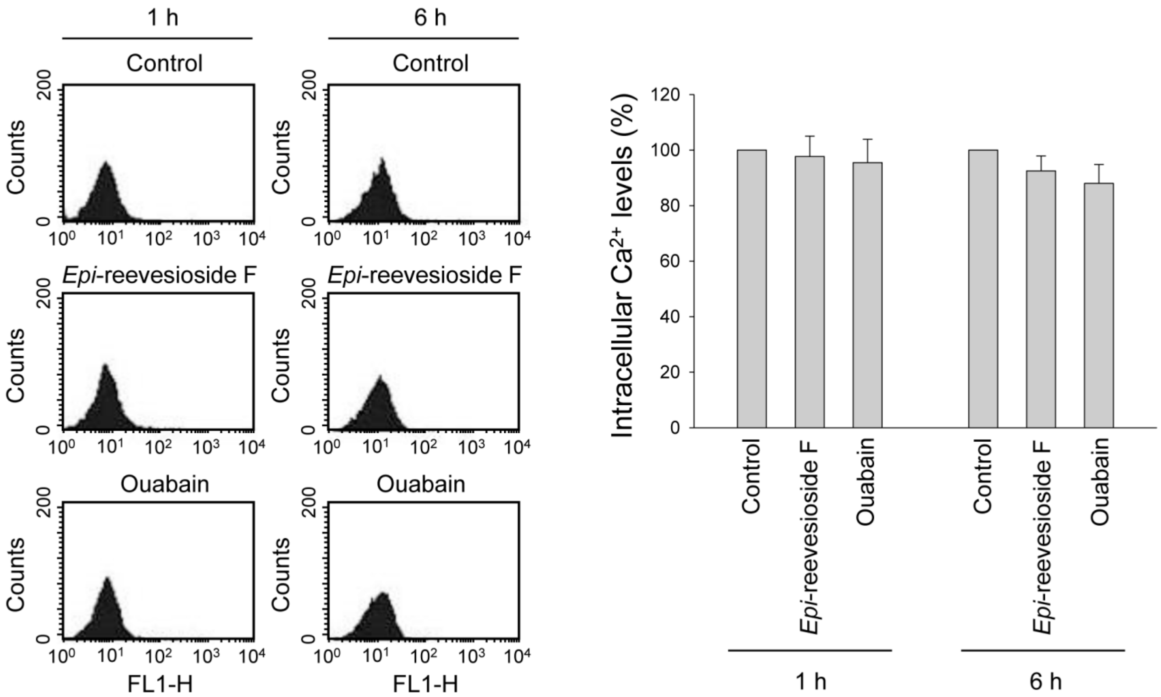

Supplementary figure 2: Effect of *Epi*-reevesioside F and ouabain on intracellular Ca^{2+} mobilization. T98 cells were incubated in the absence or presence of *Epi*-reevesioside F (1 μM) or ouabain (1 μM). The intracellular Ca^{2+} levels were measured by flow cytometric analysis. Data are expressed as mean±SEM of four determinations.

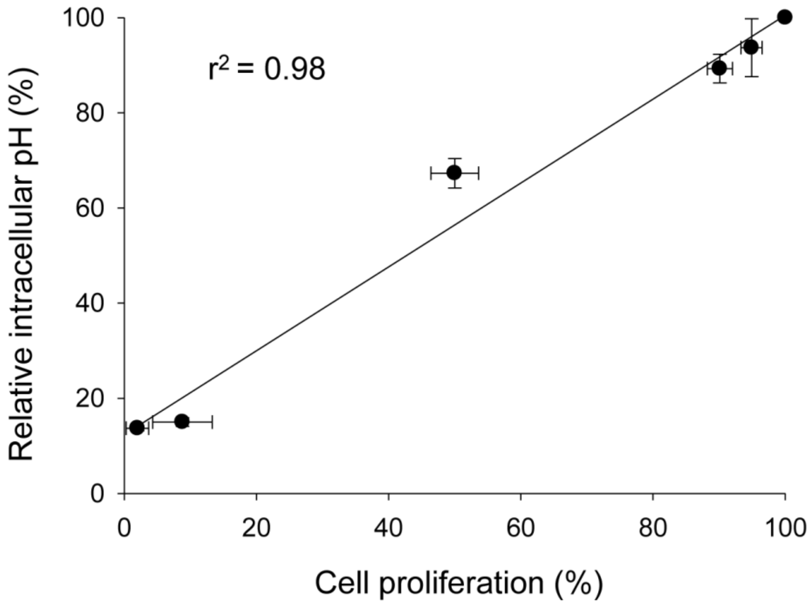

Supplementary figure 3: Correlation between the effects on anti-proliferation and intracellular acidification induced by *Epi*-reevesioside F. T98 cells were incubated in the absence or presence of *Epi*-reevesioside F. The cell proliferation and intracellular pH were examined using sulforhodamine B assay and flow cytometric analysis, respectively. Quantitative data are expressed as mean±SEM of three to four independent determinations. The correlation between two parameters was made and correlation coefficient (r^2) was calculated.

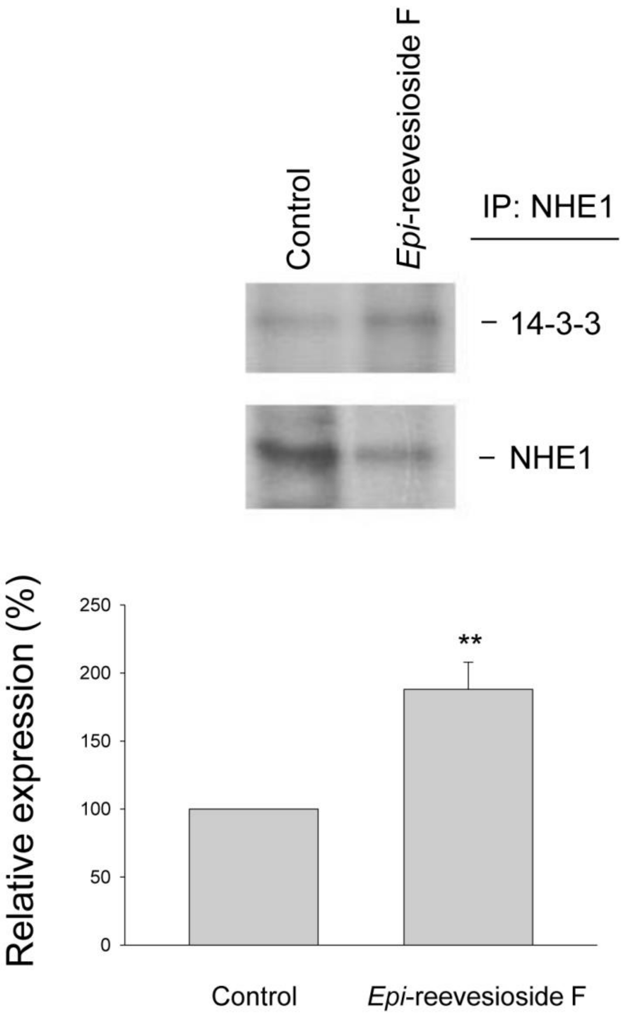

Supplementary figure 4: Effect of *Epi*-reevesioside F on the association of 140-3-3 and NHE1. T98 cells were incubated in the absence or presence of *Epi*-reevesioside F (100 nM) for 24 hours. After the treatment, the cells were harvested for the detection of protein expressions of 14-3-3 and NHE1 by immnoprecipitation and Western blot analysis. Data are expressed as mean±SEM of two independent determinations. ***P* < 0.01 compared with the control.

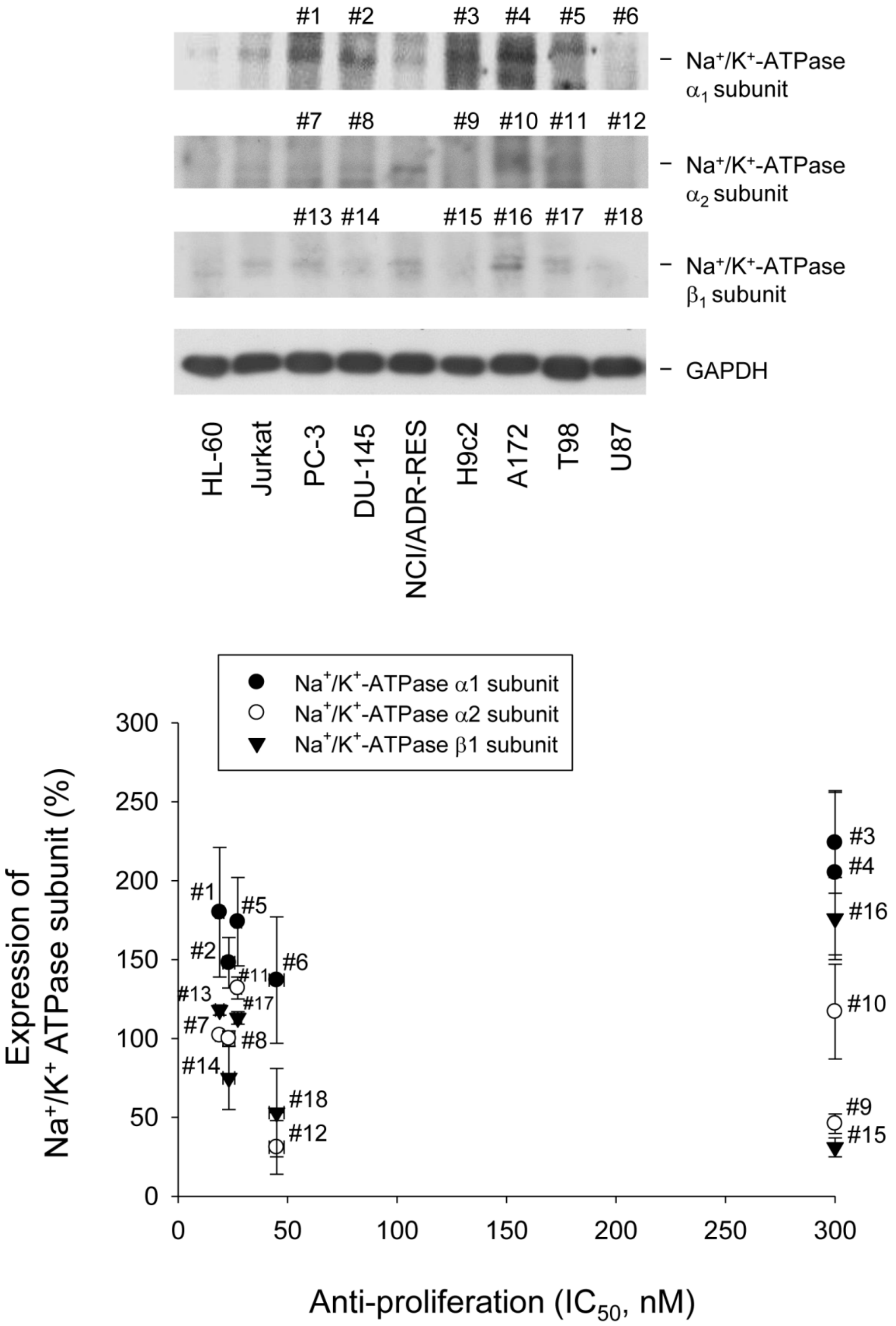

Supplementary figure 5: Protein expressions of several Na^{+}/K^{+}-ATPase subunits and correlation between protein expression of Na^{+}/K^{+}-ATPase subunit and anti-proliferative activity. The expressions of Na^{+}/K^{+}-ATPase subunits were detected using Western blot analysis. The anti-proliferative IC_{50} values were determined using SRB assays. Data are expressed as mean±SEM of three independent experiments.
